# Safety of endoclips for magnetic resonance imaging

**DOI:** 10.1002/acm2.70688

**Published:** 2026-07-06

**Authors:** Wingchi Edmund Kwok, Adam Gentile, Randy Crane

**Affiliations:** ^1^ Department of Imaging Sciences University of Rochester Medical Center Rochester New York USA

**Keywords:** endoclip, implant, image artifacts, magnetic force, magnetic resonance imaging, MRI safety

## Abstract

**Background:**

A fatal incident in which magnetic resonance (MR) may be involved and the appearance of strong MR attraction experienced by some MR‐conditional endoclips have cast confusion over the MR safety of MR‐conditional endoclips, which may lead to unnecessary delay or denial of MRI to some patients. On the other hand, there are endoclips that are MRI‐unsafe, yet not all MRI facilities screen patients for endoclips.

**Purpose:**

Our purpose is to promote a better understanding of the MRI risks associated with endoclips to facilitate proper management of MRI exams involving patients with endoclips.

**Methods:**

Deflection angle measurements were conducted on three models of MR‐conditional endoclips on a 1.5T and a 3T MRI system to assess the magnetic forces on the endoclips. Detachment force measurements were also performed on porcine stomach mucosa specimens to evaluate the attachment force. Besides, detachment tests were conducted inside the magnet bore to evaluate the effect of magnetic‐induced torque.

**Results:**

The measured detachment force was much larger than the magnetic force for each clip tested, with the ratios of the two forces being 87.3–251 at 1.5T and 46.7–130 at 3T, indicating low detachment risk for these clips. This was further supported by the detachment tests inside the magnet bore in which no endoclip detached.

**Conclusions:**

This study reinforces the MR conditional labeling on endoclips, demonstrating that the appearance of strong magnetic force does not necessarily imply an endoclip is MR unsafe. If the counter force (i.e., the detachment force) provided by an endoclip is much larger than the MRI magnetic force, as observed in this study, the risk of clip detachment during MRI is low. This paper should assist in facilitating proper management of MRI exams involving patients with endoclips, preventing unnecessary denial of MRI examinations to patients while protecting patient safety.

## 1 INTRODUCTION

**FIGURE 1:**
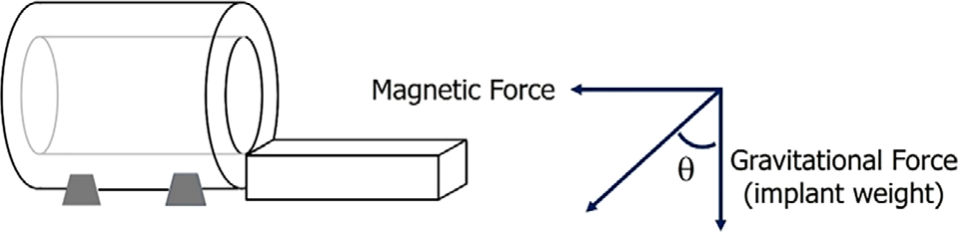
Sketch diagram showing the deflection angle measurement based on the American Society for Testing and Materials (ASTM) standard. Measurement is conducted on the bore axis at the location where the translational magnetic force is the largest. For an object sustaining a deflection angle θ, the magnetic force equals its weight multiplied by tan(θ).

Endoclips are metallic devices used in endoscopy procedures, such as perforation sealing and hemorrhage treatment, to close mucosal surfaces without surgery and stitches. They typically remain attached to the applied tissue for days to weeks until the tissue heals, after which they eventually detach and get excreted.^1^, ^2^ The safety of having endoclips in the gastrointestinal tract during magnetic resonance imaging (MRI) depends on the specific model of endoclip applied. While some endoclips are non‐ferromagnetic, many are ferromagnetic and subject to magnetic forces from the MRI magnet. Endoclips may be labelled MR‐conditional (i.e., safe for MRI under specific conditions), MR‐unsafe, or untested and unlabeled for MRI.^3^, ^4^ Unfortunately, in 2012, a patient with an MR‐conditional endoscopic clip in the esophagus underwent brain MRI and died shortly afterwards of severe esophageal bleeding.^5^ This led to the revelation that some MR‐conditional endoclips sustain large deflection angles (> 45°) near MRI magnets (Figure 1) ^6^, ^7^ or even “fly” into the magnet (See video in Supporting Material), seemingly indicating strong magnetic attraction. These facts, combined with common warning comments about potential bleeding in their MR safety labeling, have cast confusion in the MR community over the safety of MR‐conditional endoclips, which may lead to unnecessary delay or denial of MRI to patients with endoclips. On the other hand, some endoclips are MR unsafe,^8^, ^9^, ^10^, ^11^ yet it has been reported that not all MRI facilities screen patients for endoclips.^8^ The purpose of the current study is to conduct a relevant MR safety evaluation on ferromagnetic endoclips to demonstrate the rationale for their MR conditional designation and promote a better understanding of the depth of assessment that goes into an MR safety label designation, and hopefully promoting timely access for those patients with MR conditional clips needing MRI.

## 2 METHODS

Three MR‐conditional endoclips commonly utilized at our medical center were evaluated in this study, namely Resolution 360 (Boston Scientific, Massachusetts, USA), InstinctPlus (Cook Medical, Indiana, USA), and DuraClip (CONMED, Florida, USA) (Table 1). For each endoclip, deflection angle measurement was conducted to assess the MRI translational magnetic force ^6^, ^9^ while detachment force measurement was performed to assess the attachment force of the endoclip.^9^ Detachment tests were also performed inside the magnet bore to observe for clip detachment under the influences of both the magnetic‐induced torque and the translational magnetic force, as described in another study.^10^

**TABLE 1:** The three evaluated endoclips and their relevant labeling information.

	Resolution 360™ 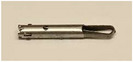	Instinct Plus 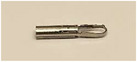	DuraClip 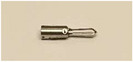
Manufacturer	Boston Scientific	Cook Medical	ConMed
Model Number	M00521230	G58010	DC0235
Material (According to information provided by the manufacturer)	Stainless steel 73% Cobalt‐chrome 24% Styrene acrylonitrile copolymers 3%	Stainless‐steel, Delrin, and Nitinol	Stainless‐steel with traced amount of nickel
MR‐conditional? Static magnetic field in MR safety label	Yes, 1.5T and 3T	Yes, 1.5T and 3T	Yes, 1.5T and 3T
Maximum SFG in MR safety label (G/cm)	3000	1900	4000
Length (mm)	16.5	14.4	10.0
Opening width (mm)	11	16	11
Weight (gram)	0.112	0.169	0.114

**FIGURE 2:**
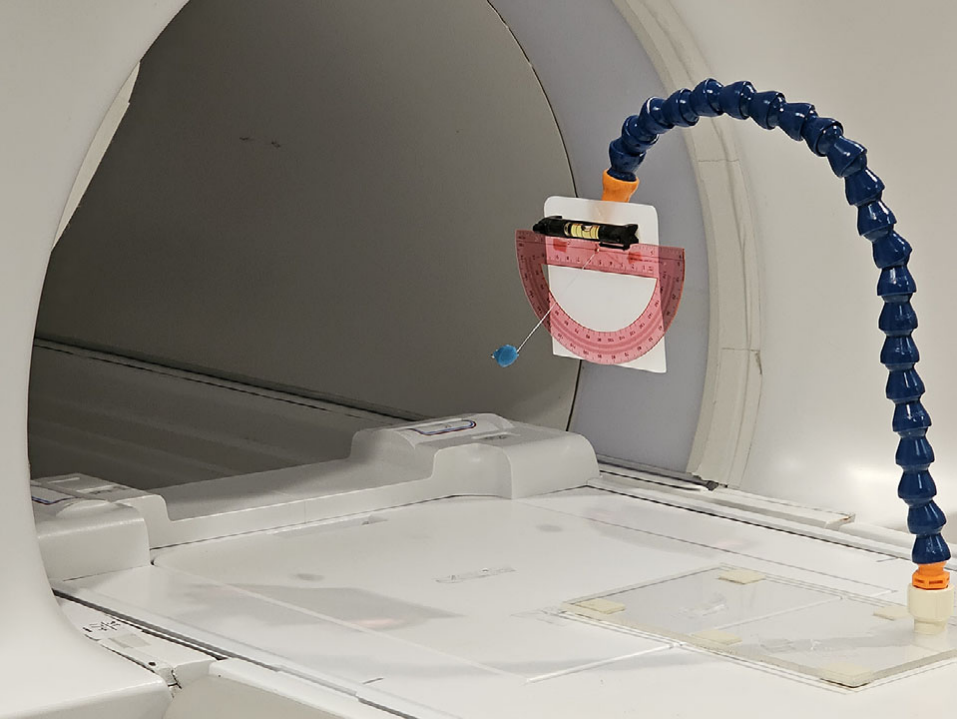
Deflection angle measurement setup in this study. Each endoclip was attached to the end of a dental floss on a home‐built device. Modeling clay was added to lower the deflection angle to 45° at which the combined weight of each endoclip and its corresponding modeling clay equaled the magnetic attraction force experienced by the endoclip.

The deflection angle measurement was conducted on both a General Electric Artist 1.5T system (GE HealthCare, Illinois, USA) and a General Electric Premier 3T (GE HealthCare, Illinois, USA). Using a deflection angle measurement setup (Figure 2), the translational magnetic attraction force experienced by each endoclip was measured. Based on the spatial field gradient (SFG) information provided by GE HealthCare for the two MR systems, each endoclip was placed at the maximum SFG location along the central axis of the magnet bore (following the ASTM F2052 guideline on Measurement of Magnetically Induced Displacement Force on Medical Devices in the MR Environment).^6^ This location was chosen since the endoclips are likely magnetically saturated near the MRI magnet, which was later confirmed by the test results. The measured translational magnetic force was thus proportional to the SFG only and not related to the MR system magnetic field.^7^ Each endoclip was attached to the end of a dental floss on the device. As all evaluated endoclips demonstrated deflection angles much larger than 45°, to enable more accurate force measurement, modeling clay (tested with a hand‐held ferromagnetic detector to confirm being non‐ferromagnetic) was added near each endoclip in an amount to lower the deflection angle to 45°. The vertical position of each clip was also measured using a non‐ferromagnetic tape measure and adjusted to 34 cm from the top of the 70 cm (measured 68 cm) bore. At the deflection angle of 45°, the combined weight of each endoclip and its corresponding modeling clay, measured using an electronic balance (VWR, Pennsylvania, USA), represented the magnetic attraction force experienced by each endoclip. For each clip, the deflection testing device was then moved right/left, in/out and up/down by several centimeters to check for any changes in the deflection angle. Following ASTM F2052 guideline, each measurement was also repeated two times with the device being moved away and then placed back to the marked location on the scanner table to check for repeatability.

**FIGURE 3:**
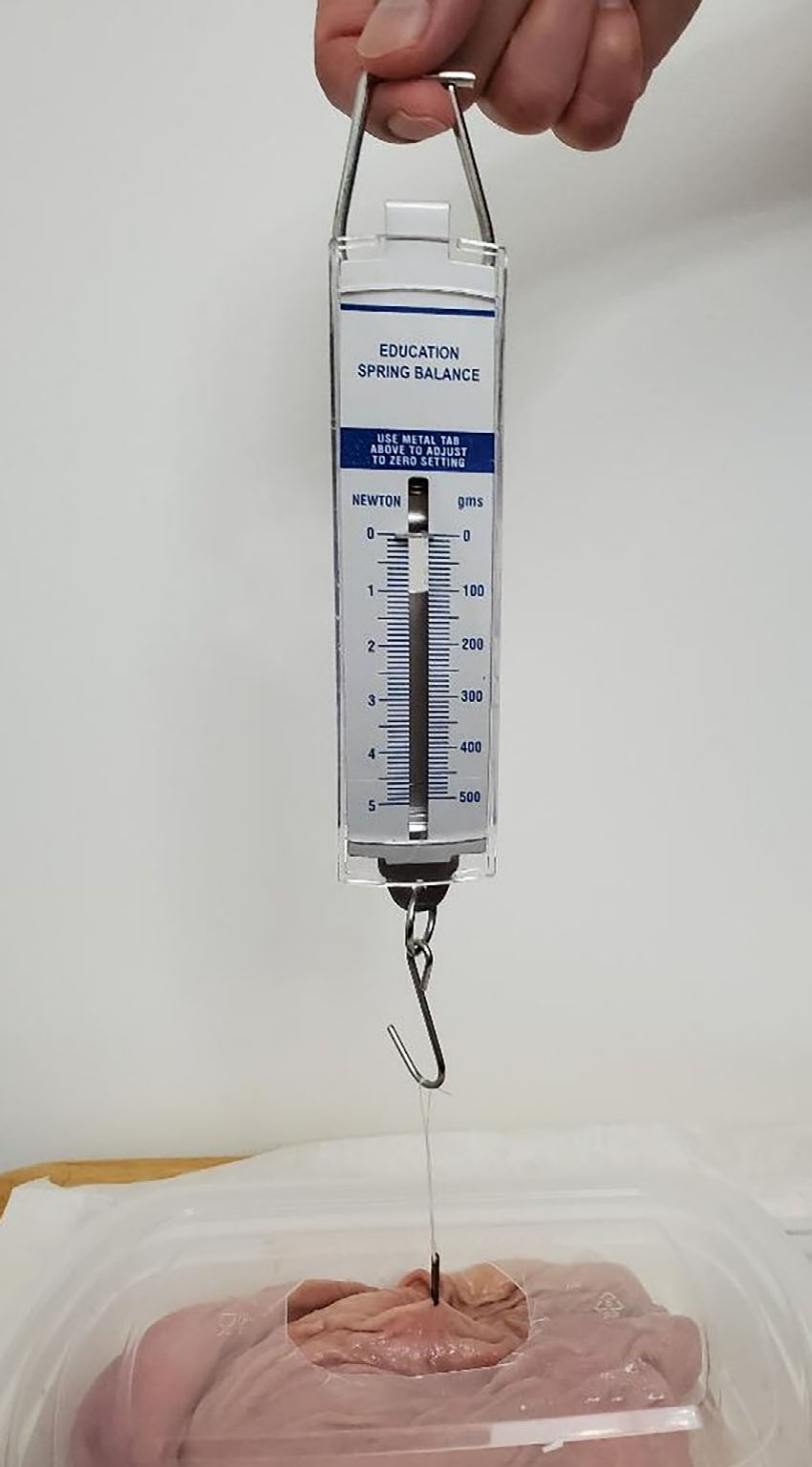
Detachment force measurement setup in this study. Each endoclip was attached to a piece of pig stomach mucosa specimen and connected to a calibrated spring balance via dental floss. The detachment force equaled the reading of spring balance when the endoclip was completely detached from the tissue while the spring balance was slowly raised.

For the detachment force measurement, each endoclip was attached to a piece of porcine stomach mucosa specimen and connected to a calibrated spring balance via dental floss (Figure 3). As the spring balance was slowly raised, the balance's reading at the point when the endoclip detached was recorded as the detachment force. The measurement was repeated two times for each type of endoclips using a different clip unit and a different porcine stomach specimen.

The measured magnetic force and detachment force for each endoclip were then compared. A detachment force much larger than the magnetic force would indicate a low likelihood of detachment due to the force on the clip from MRI.

For the detachment test inside the magnetic bore, three endoclips, one from each clip type, were attached to a porcine stomach in a plastic container and separated from each other by about 1.5 cm. The setup was placed centered (left/right) on the scanner table, moved into the magnet from the landmark position, passed the magnet isocenter, and then moved back to the landmark position. The test was performed at three orientations – 0°, 45°^,^ and 90° with respect to the magnet bore direction. The experiment was then repeated by shifting the setup 15 cm laterally and tested at the three orientations. This test was conducted on both the 1.5T and the 3T systems.

## 3 RESULTS

The weight of each endoclip and that of the modeling clay added to reduce the deflection angle to 45°, as well as the calculated magnetic force and the measured detachment force, are listed in Table 2. The ratios of the average detachment force to the translational magnetic attraction force at 1.5T and 3T were found to be 155 and 78.5 for Resolution 360, 87.3 and 46.7 for Instinct Plus, and 251 and 130 for DuraClip respectively.

**TABLE 2:** Results of the detachment force and translational magnetic force measurements.

Endoclip type (Model number)	Resolution 360 (M00521230)	Instinct plus (G58010)	DuraClip (DC0235)
Detachment force measurement			
Test 1/ Test 2 / Test 3 (gram) Average (gram)	240/200/180 207	280/230/260 257	220/155/160 178

Magnetic force measurement						
Clip weight (gram)	0.112		0.169		0.114	
	1.5T	3T	1.5T	3T	1.5T	3T
Added modeling Clay Weight (gram)	1.221	2.526	2.775	5.329	0.595	1.258
Magnetic force (gram) = Weights of clip + modeling Clay	1.333	2.638	2.944	5.498	0.709	1.372
Force ratio (detachment / magnetic)	155	78.5	87.3	46.7	251	130

Table 3 shows that the ratio of the magnetic force experienced by each endoclip between the 1.5T and 3T scanners was the same as the ratio of the SFG at the points of measurement between the two systems, indicating that the magnetic force was proportional to the SFG. Since the translational magnetic force is proportional to the magnetic field * SFG, the fact that our measured magnetic force was proportional to SFG only but not to the MR system magnetic field implies that the endoclips were magnetically saturated at/before 1.5T. Since the location of measurement was at the maximum SFG along the bore axis, the translational magnetic force was also at its maximum, which was confirmed by moving the measurement device in and out by several centimeters and observing the accompanied decrease in deflection angle. When the position of each clip was moved by several centimeters laterally and up/down, only minor changes in the deflection angle (< 1°) were observed. These observations are in line with the SFG maps of both systems that show relatively uniform SFG in the close neighborhood of the maximum location. Furthermore, when the whole deflection setup was moved away and then placed back to the marked location of measurement, no change in the deflection angle was observed, indicating high repeatability of the measurement.

**TABLE 3:** Comparison of the magnetic force ratio and the SFG ratio at the maximum SFG location along the bore axis between the 3T and the 1.5T systems.

Endoclip type (Model number)	Resolution 360 (M00521230)	Instinct plus (G58101)	DuraClip (DC0235)
Magnetic force at 3T	2.638	5.498	1.372
Magnetic force at 1.5T	1.333	2.944	0.709
Magnetic force ratio (3T/1.5T)	1.979	1.868	1.935
SFG at 3T	556	556	556
SFG at 1.5T	291	291	291
SFG Ratio (3T/1.5T)	1.91	1.91	1.91
Percentage differences between magnetic force ratio and SFG ratio	+3.61%	−2.20%	+1.31%

In the detachment test inside the magnet bore of both the 1.5T and 3T systems, none of the three endoclips detached while moving in and out of the scanner at different locations and orientations on the scanner table.

## 4 DISCUSSION

In this study, though all three MR‐conditional endoclips demonstrated ferromagnetic displacement force greater than their weight, the high ratios of the detachment force to the magnetic attraction force indicate low detachment risk. This was further supported by the detachment test inside the magnet bore in which none of the three endoclips detached at both 1.5T and 3T. Furthermore, the magnetic attraction force experienced by each endoclip on the 1.5T system was about half that on the 3T system due to the lower SFG of the 1.5T system, indicating potentially lower risk for systems with lower SFG values that the patient may experience during MRI.

An earlier study investigating two endoclips ^9^ found that a non‐MR‐conditional endoclip (EZ clip from Olympus Medical System, Korea) did not detach nor cause tissue damage at 3T though it displayed ferromagnetism. On the other hand, another study evaluating four different types of endoclips at 1.5T ^10^ found one model (TriClip from Cook Endoscopy, Winston‐Salem, NC) detached from gastric tissue and thus was deemed unsafe for MRI. The other three types did not show detachment.

The endoclips evaluated in this study include Instinct Plus, a newer model replacing Instinct that was involved in the fatal incident ^5^ but is no longer produced by the manufacturer. In that incident, the endoclip was applied to a long and deep laceration in the esophageal wall six days before the MR exam, probably not giving the site tissue sufficient time to heal after surgery to reduce/prevent the injury when the endoclip detached. Serious tissue damages such as this might need longer time to heal while also weaken the clip attachment and result in serious consequences when detachment occurs. However, it is also possible that the fatal incident was coincidental and not caused by exposure to the MR system.

Though tissue damage or perforation is also a potential concern, the force required to generate bowel perforation by the clip is expected to be much greater than the force needed to detach the clip.^9^

While most endoclips fall off by itself within 3–4 weeks, some can remain at the site of application for up to 1 year ^1^ and so it may not be possible/desirable to delay an MRI exam until all the endoclips pass. Thus, it is important to have a good understanding of the MRI risk associated with endoclips in order to not unnecessarily delay the MRI exam. As demonstrated in this study, the appearance of strong magnetic force, for example, displaying larger than 45° in the deflection test, does not necessarily imply an endoclip is MR unsafe. The counter force (i.e., detachment force) provided by the endoclip may be much larger than the MRI attraction force,^7^ as observed in this study and reported in other studies,^9^, ^10^, ^11^ preventing the clip from being moved or dislodged and allowing a patient to undergo MRI safely for a properly implanted endoclip. In addition, the light weight of each endoclip in this study (less than 0.2 grams) implies that a greater‐than 45° deflection itself may not necessarily represent a strong magnetic attraction force that can cause direct harm to the neighboring tissue.

In a Canadian study,^8^ 24% of MR sites surveyed did not screen patients for endoclips. Since some endoclips are MRI‐unsafe, there is a need to screen MRI patients for endoclips.^8^, ^9^, ^10^, ^11^ Patients who have undergone recent endoscopy or colonoscopy (e.g., within the prior 8 weeks as in the MR safety policy of our institution) should be checked for the placement of endoclips. If there are endoclips placed inside the patient's body, the type of endoclips should be identified to determine MR safety, for example, from implant card or surgical note. For MR‐conditional endoclips, patients may safely undergo MRI if the MR safety conditions provided by the endoclip manufacturer are met.^3^, ^4^ If the endoclip is known to be made of titanium, which can be confirmed by its manufacturer, it is also safe to scan. However, if the endoclip is MR‐unsafe, unlabeled/type unknown or unclear whether a clip was placed, an abdominal X‐ray may be needed to confirm passing/absence of endoclips. If the endoclip remains in the body, it may require the radiologist to conduct risk‐benefit analysis to determine whether to proceed with the MRI or have the MRI delayed until the endoclip passes.

Radiofrequency (RF) heating is not expected to pose significant MRI risk to endoclips due to their small sizes. According to an FDA guideline on medical devices,^4^ “A passive implant with dimensions of less than 2 cm in all directions and at least 3 cm away from another passive implant does not need to be tested with respect to RF induced heating at 3.0 T or less, as it is expected to generate a temperature increase of less than 2°C in Normal Operating Mode, over the course of 1 h of exposure”. Similarly, time‐varying magnetic field gradient used for spatial encoding is also not a concern for endoclips, as the same FDA guideline states that due to the typical small planar surface area of passive medical devices, gradient induced heating and gradient induced vibration are generally not expected to pose a hazard for tissue damage.^4^

**FIGURE 4:**
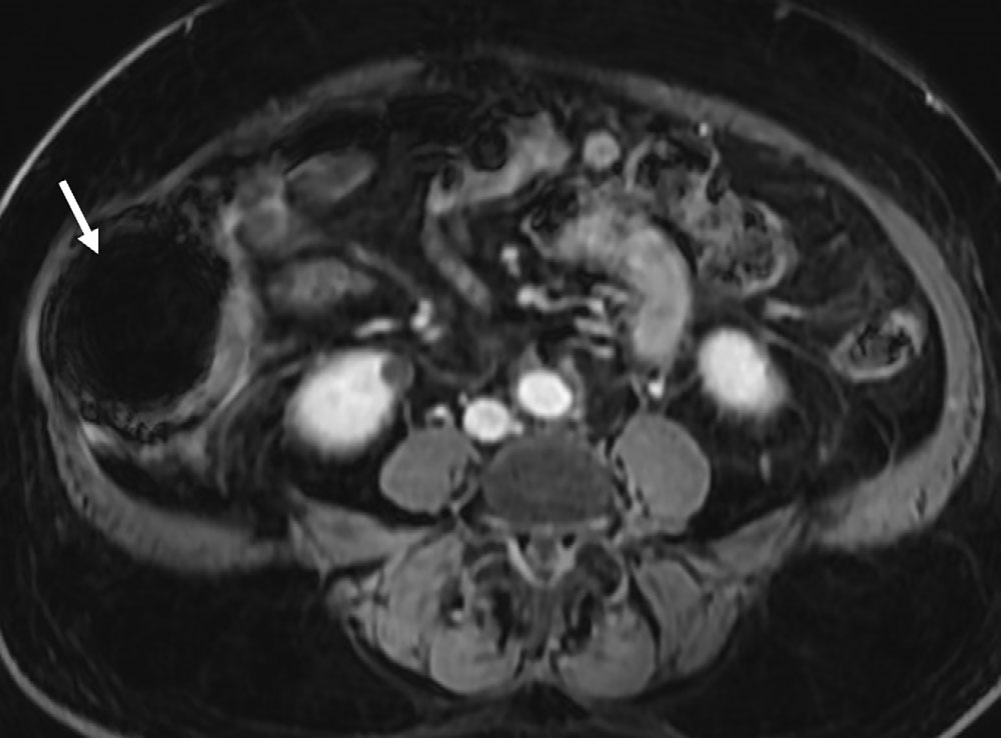
Signal void image artifact (arrow) caused by the presence of MR‐conditional endoclips.

Besides safety concerns, image artifact caused by MR‐conditional endoclips is also a factor to consider when imaging nearby tissues. As shown in Figure 4, the magnetic susceptibility ^12^ of endoclips may cause signal void and image distortion in the surrounding tissues as well as fat suppression issues.

There are limitations of this study. One is that the detachment experiments were only conducted on healthy porcine stomachs, which might not accurately represent human organs and diseased/damaged (inflamed, ulcerated, or scarred) tissues. In addition, only three types of endoclips were evaluated. Future studies will benefit from including both healthy and diseased/damaged tissues as well as a larger variety of endoclips.

## 5 CONCLUSION

This study reinforces the MR conditional labeling on endoclips, demonstrating that the appearance of strong magnetic force does not necessarily imply an endoclip is MR unsafe. If the counter force (i.e., the detachment force) provided by an endoclip is much larger than the MRI magnetic force, as observed in the three endoclips in this study, the risk of clip detachment during MRI is low. This paper should assist in facilitating proper management of MRI exams involving patients with endoclips, preventing unnecessary denial of MRI examinations to patients while protecting patient safety.

## AUTHOR CONTRIBUTIONS

Wingchi E Kwok: Conception and design of the study, methodology, analysis, and writing. Adam Gentile: Methodology and writing. Randy Cane: Conception of the study and writing.

## CONFLICT OF INTEREST STATEMENT

The authors have no relevant conflicts of interest to disclose.

## SUPPLEMENTARY MATERIAL

Video of an MR‐conditional endoclip “flying” into a 3T MRI magnet.

## Supporting information




**Supporting File**: acm270688‐supp‐0001‐movie.mp4.

## Data Availability

The data that support the findings of this study are available from the corresponding author upon reasonable request.
